# The Arousal Effect of Exclusionary and Inclusionary Situations on Social Affiliation Motivation and Its Subsequent Influence on Prosocial Behavior

**DOI:** 10.3389/fpsyg.2021.594440

**Published:** 2021-03-02

**Authors:** Esther Cuadrado, Carmen Tabernero, Antonio R. Hidalgo-Muñoz, Bárbara Luque, Rosario Castillo-Mayén

**Affiliations:** ^1^Maimonides Biomedical Research Institute of Córdoba (IMIBIC), Córdoba, Spain; ^2^Department of Psychology, University of Córdoba, Córdoba, Spain; ^3^Department of Social Psychology, Instituto de Neurociencias de Castilla y León (INCYL), University of Salamanca, Salamanca, Spain; ^4^CLLE, UMR 5263, CNRS, University of Toulouse Jean-Jaurès, Toulouse, France

**Keywords:** social exclusion, skin conductance, emotional state, social affiliation motivation, prosocial behavior

## Abstract

Given the negative costs of exclusion and the relevance of belongingness for humans, the experience of exclusion influences social affiliation motivation, which in turn is a relevant predictor of prosocial behavior. Skin conductance is a typical measure of the arousal elicited by emotions. Hence, we argued that both inclusion and exclusion will increase skin conductance level due to the increase of either positive affect or anger affects, respectively. Moreover, we argued that emotional arousal is also related to social affiliation motivation and prosocial behavior. A total of 48 students were randomly allocated to either an inclusionary or exclusionary condition and their skin conductance levels were recorded during an experiment in which they completed an online questionnaire and played the game “Cyberball.” Results indicated that (a) individuals who perceived high exclusion felt angrier than individuals perceiving high inclusion, who feel positive affect; (b) no differences were evidenced in terms of skin conductance between exclusion and inclusion situations; (c) over-aroused individuals were less motivated to affiliate; and (d) individuals with lower affiliation motivation behaved in a less prosocial way. The results were congruent to the argument that behaving prosocially may be a way to gain the desired affiliation.

## Introduction

People are inherently motivated to maintain connections and to belong to a group (Baumeister and Leary, 1995). Individuals are social creatures and communities provide them with a sense of belonging, an identity, and meaning in their life (Butler et al., 2007). Furthermore, social acceptance becomes even more important for youths, who, being immersed in their identity creation process, attach greater importance than adults to feeling included in a group. In contrast, social isolation and the lack of strong social bonds are linked to poor mental, physical, and psychological health and a higher death rate (House et al., 1988; Bugental, 2000; Cacioppo et al., 2003; Cacioppo and Patrick, 2008; Gable and Impett, 2012).

There is a traditional controversy about whether social exclusion leads to low or high social affiliation motivation, and different studies have evidenced in which circumstances excluded individuals’ affiliation motivation will increase or decrease (Maner et al., 2007; Romero-Canyas et al., 2010; Molden and Maner, 2013; Cuadrado et al., 2015). However, some questions remain unexplored regarding how individuals react to situations of social exclusion that offer the possibility of developing prosocial or antisocial behaviors (Debono et al., 2020). The social connection hypothesis has been supported, and it seems that rejected individuals try to reconnect with individuals who have not rejected them or with whom future interaction is expected, but not with those who have rejected them, with no future interaction being expected, and with whom the perception of future reconnection is impossible (Maner et al., 2007; Molden and Maner, 2013). Moreover, exclusion usually triggers emotions that lead to anger (Cuadrado et al., 2015), and anger has been related to more antisocial behavior (Chow et al., 2008).

Nonetheless, to our knowledge, the physiological correlates of the emotions elicited by exclusion and inclusion situations has scarcely been taken into consideration in previous studies. However, some findings suggest that being excluded or ostracized interacts with specific body manifestations, such as skin temperature, as shown by IJzerman et al. (2012) in their study, where exclusion was associated with lower skin temperature and alleviated with warm drinks. Thus, together with the subjective feelings explained above, the intensity of these affective reactions in terms of body reactions or physiological arousal may also impact the subsequent social affiliation motivation.

### Effects of Exclusion/Inclusion Situations on Perceived Subjective Emotion (H1) and Emotional Arousal (H2)

Social exclusion has been seen to cause emotional distress, pain, and negative moods and to be associated with psychological problems (Williams et al., 2000; Deater-Deckard, 2001; Leary et al., 2001; Nolan et al., 2003; Buckley et al., 2004; Gonsalkorale and Williams, 2007; Williams, 2007). Social exclusion has also been identified as activating neural pathways associated with pain and distress (Eisenberger and Lieberman, 2004; Eisenberger et al., 2007, 2011; Kawamoto et al., 2012; Sleegers et al., 2017). The emotional reaction to social exclusion has been seen to be immediate (Williams et al., 2000, 2002; Gonsalkorale and Williams, 2007) and generates a significant decrease in positive affect in parallel with a significant increase in anger (Seidel et al., 2013). In contrast, feeling included in a group enhances positive affect in individuals (Blackhart et al., 2009; Cuadrado, 2015; Cuadrado et al., 2015). Thus, as hypothesis 1, we expected social exclusion to lead to higher levels of anger and lower levels of positive affect in comparison with social inclusion. This means that an exclusion situation will enhance anger levels in comparison with an inclusion situation, whereas an inclusion situation will enhance positive affect in comparison with an exclusion situation (Hypothesis 1a).

Moreover, given the impact of exclusion on individuals as a very stressful and threatening factor (Eisenberger and Lieberman, 2004) and in agreement with the negative bias found in several studies, where negative emotions are more prominent (e.g., in Cacioppo et al., 1999; Carretié et al., 2001), we expected the variations in anger during inclusion or exclusion situations will be higher than the variations in positive affect (Hypothesis 1b).

When individuals perceive inclusion cues, they react emotionally with an increase in positive affect and a decrease in negative moods (Blackhart et al., 2009; Cuadrado et al., 2015), whereas when individuals perceive exclusion cues they react with more negative emotions, such as anger (Chow et al., 2008; Romero-Canyas et al., 2010; Cuadrado et al., 2015). Both experiences—inclusion and exclusion—will not only elicit subjective feelings but also physiological changes closely linked to these feelings. As is well known, elicitation of emotion induces autonomic nervous system responses; and one of the most reliable measures to detect physiological activation is skin conductance (Boucsein, 2012). Thus, as we have argued before, situations that induce intense emotions—such as exclusion or inclusion—should also activate the neural autonomic pathway and this reaction may be measured via the skin conductance response. Therefore, we expected both situations would impact on skin conductance due to the increase in arousal of the emergent emotions, disregarding their affective valence. Hence, in agreement with our previous hypothesis concerning perceived subjective emotions, we expected that the exclusion condition will elicit higher emotional arousal than inclusion situations (Hypothesis 2).

### Relationship of Skin Conductance on Social Affiliation Motivation and Prosocial Behavior in Excluded Versus Included Individuals (H3)

Given the negative costs of social exclusion and the key role by belongingness in human functioning, the feeling of social exclusion may foster the desire for individuals to reconnect, to gain acceptance, and to be accepted and included in a group in order to reduce the pain and other aversive reactions provoked by social exclusion (DeWall et al., 2011). As DeWall (2010) has demonstrated, when forming attitudes, people who feel socially excluded are particularly influenced by individuals who are a potential source of inclusion and affiliation, which shows that social exclusion perception increases the desire for social connection. In other words, given the importance of belongingness for the survival and well-being of individuals, the painful experience of social exclusion often leads individuals to try to reconnect with others (i.e., higher levels of social affiliation motivation) in an attempt to repair their feelings of social acceptance (Gardner et al., 2005; Pickett and Gardner, 2005; DeWall, 2010; DeWall et al., 2011; Molden and Maner, 2013). In contrast, the desire to reconnect should not arise in included individuals; this would be pointless because their inclusion is currently secure (Cuadrado, 2015). Nevertheless, rejected individuals may be motivated to affiliate only with others who have not rejected them, avoiding individuals and groups that have rejected them and with whom no face-to-face interaction is expected (Maner et al., 2007).

In this sense, we can easily imagine that the negative affect induced by rejection will result in a lower affiliation motivation with the rejecters, in line with the theory developed by Maner et al. (2007). In the same line, Serafini et al. (2017) have found that individuals with extreme sensory sensitivity—who are hypersensitive to rejection—also show exaggerated negative emotionality and high tendency to depression. Then, bearing in mind both the social reconnection hypothesis (Maner et al., 2007) and the Cognitive Affective Personality System (Mischel and Shoda, 1995; Mendoza-Denton et al., 2001) which suggest that individuals’ interpretations of situations impact their reactions, outcomes, and behaviors, we can also easily imagine that for individuals who react strongly to rejection (over-arousal), their need to belong to the group of individuals who have rejected them will decrease. However, for individual who and react strongly to inclusion, no relation between skin conductance and social affiliation is expected, since their need to belong is already satisfied.

Then, we expect that skin conductance will be related with lower levels of social affiliation motivation in the excluded group (Hypothesis 3a). Furthermore, a positive correlation between prosocial behavior and skin conductance response has been found (Hein et al., 2011). In their study, the authors suggest that skin conductance level is linked to empathy and correlates with the costly helping tendency to prevent others from feeling pain. Thus, we can expect a positive correlation between the level of arousal (skin conductance) and the prosocial behavior in the inclusion condition and an inverse pattern for the exclusion condition (Hypothesis 3b).

### The Social Affiliation Motivation–Prosocial Behavior Link (H4)

Individuals need to maintain social bonds and acting in a prosocial manner with others may be a way towards achieving this. Nevertheless, to the best of our knowledge, the relationship between affiliation motivation and prosocial behavior has not been sufficiently studied. Boyatzis (1973) affirmed that high levels of affiliation motivation make individuals genuinely interested in others. Furthermore, it has been shown that individuals with high affiliation motivation levels tend to react to other people in a friendly way and to be altruistic (Baumeister and Leary, 1995; Mlcak and Zaskodna, 2008) and prosocial (Cuadrado et al., 2015) in order to maintain or create social bonds. Moreover, Cuadrado et al. (2015) argued that social affiliation motivation is a highly relevant variable in the prediction and fostering of prosocial behavior, suggesting that this variable may be a powerful predictor of prosocial behavior in both included and excluded individuals. Thus, we expected that high levels of affiliation motivation would predict higher levels of prosocial behavior; in order to fulfill their need for social contact, individuals who are very motivated to be affiliated with a group will tend to behave prosocially with this group in order to be accepted, to ensure their inclusion and maintain social contact and social acceptance in the group with which future interaction is expected (Hypothesis 4).

## Materials and Methods

### Participants

Fifty youths with normal or corrected-to-normal vision took part in the study. Participants were recruited using advertisements at the Faculty of Social Sciences of the University of Córdoba (Spain). Because the majority of students were women in the educational sector, only women responded to the call, and so the sample was exclusively comprised of women. Data from two participants were removed because the software failed in their sessions, leaving data from 48 participants (age range 18–22; mean age = 19.56) in the final analysis. Participants were randomly allocated to the experimental conditions of exclusion (24 participants) or inclusion (24 participants).

The study was conducted with the approval of the Andalusian Biomedical Research Ethics Committee. The procedure was fully explained to participants; they were aware that participation was voluntary and that they could choose to discontinue at any time. Finally, data were analyzed anonymously.

### Procedure

The procedure was the same as that of Cuadrado (2015). Participants individually arrived at the laboratory, where they stayed for approximately one hour to complete all the tasks. To avoid participants becoming suspicious about the experimental manipulation, the research assistant explained to them that the study’s main objective was to analyze whether certain personal variables—dispositional, motivational, and physiological—are related to the performance of individuals within the group. They were informed that, for this purpose, their skin conductance would be recorded and that they would also be asked to complete a questionnaire and to perform several online group tasks—with online participants from other Spanish universities—that would give them the chance to earn points exchangeable for cash, with a maximum total profit of 16 euros, depending on how they performed the online group tasks. In order to increase the credibility of the study, the experimenter explained to them that they would have to introduce themselves to the other participants and that they had the possibility of taking a photograph that participants were able to see, alongside a description thereof. When participants agreed to this, the experimenter took the photograph and told them that she would submit it to the platform; meanwhile, the participant entered the online platform and completed several individual tasks.

Afterwards, skin conductance response leads were positioned on participants by the research assistants. To assess skin conductance, 9 mm electrodes were attached to the medial phalangeal surfaces of the middle and index fingers of the non-dominant hand. A layer of isotonic electrolyte gel was placed on the electrodes to increase conduction. At this time, the skin conductance response was continuously recorded throughout the experiment using a second computer connected to a biofeedback pack. The experimental design was created over three blocks where each period was assumed to contain relatively constant processes (Figner et al., 2009), therefore skin conductance response was operationalized by taking the average of several discrete measurement points distributed across these three periods: block 1 – baseline; block 2 – Cyberball manipulation time; and block 3 – information manipulation time.

Subsequently, participants entered the online platform to complete different study tasks. The platform informed them about the study and the monetary compensation (they were informed that they would do different group tasks and that their performance in the group task would enable them to gain an amount of money, with a maximum total amount of 16 euros). Participants then completed an online questionnaire in which several socio-demographic and other study variables were assessed. Before the group tasks, positive affect and anger were assessed. Then, to ensure the reliability of the online group tasks, the program asked the participants to introduce themselves to the rest of the online contestants. In order to get to know the other participants in their group and to increase the ecological validity of the experiment, they then read the descriptions of six participants (all the participants read the same descriptions of nonexistent online participants) presented with their photographs and names. At that point, they were informed that the computer had randomly incorporated them into a three-person online group. The non-real participants that appeared on the screen as their group teammates were a boy and a girl—the same for all the participants. Once the computer informed them that their online group was formed, they were told that they would now start the different group tasks, with varying rounds for each. The first group task was done in order to create the two different experimental conditions; participants played a round of the fourth version of Cyberball (Williams et al., 2012), a program created for use in research on exclusion. Participants randomly played in an exclusion condition (receiving the ball only twice) or an inclusion condition (receiving the ball ten times); in total, the game comprised 30 passes between the three players. In the game, the nonexistent members of the groups were depicted as the pictures the participants saw when they read their personal descriptions, in order to increase the ecological validity of the experiment and to cause more emotional involvement (Iffland et al., 2014). The pictures and names of the participants were those they had selected to form their group task.

Afterwards, to ensure the reliability of the second manipulation, they were asked to explain why they had thrown the ball a lot or a little to each of the two members of their group, selecting different options (e.g., because I liked/didn’t like his personal description). Next, on their screen they received manipulated information that showed the number of times their teammates had sent them the ball and why. The participants belonging to the inclusion condition received the information that they had been sent the ball many times because their teammates liked them, whereas the participants belonging to the exclusion condition received the information that they had been sent the ball only a few times because their teammates did not like them. At that point, a manipulation check was performed, by measuring their perception of exclusion and inclusion. Then, positive affect, anger, and social affiliation motivation were assessed.

At that point, participants were informed that they would continue with the second group task (the dilemma game we used to measure prosocial behavior), and later with other group tasks. They were informed that after playing the first three rounds of the dilemma game, and before the next group task, all participants would see the responses of their group members to the three rounds of the dilemma game in order to provide transparency regarding the later division of the points (exchangeable by cash) between the group members. In fact, this was done in order to manipulate their perception of the possibility of being reincluded in the group after being pro- or anti-social. People are prosocial with their rejecter in order to be reincluded. As a result, they are usually more prosocial when the potential reincluders are able to see that they are behaving in a prosocial way (Williams and Sommer, 1997). Moreover, it has been found that people are more prosocial after rejection only when they expect to interact with the target of the prosocial behavior in the future, but not when no interaction is expected (Maner et al., 2007; Molden and Maner, 2013). Subsequently, they played three rounds of the dilemma game we used to measure prosocial behavior. When they finalized the three rounds of the dilemma game, they were fully debriefed and paid. The experimenter explained to them the real goal of the study and the experimental manipulation. Additionally, they were informed that because all the tasks were simulated, they did not really play with other participants, and they agreed to the monetary solvency of the group team; the compensation was 2 euros for each participant.

### Measures

All measures, manipulations, and exclusions in the study are reported.

#### Manipulation Check: Perception of Exclusion Scale

After the experimental manipulation, a manipulation check was performed. Perceptions of exclusion (α = 0.93) were measured with the four items used by Cuadrado (2015): “My group members have excluded me”; “My group members have included me” (reversed); “I feel excluded by my group members”; and “I feel included by my group members” (reversed). The levels of positive affect and anger were measured before and after the exclusion/inclusion manipulation in order to assess whether the manipulation had any effect on it. Moreover, the skin conductance response was recorded before playing Cyberball, during Cyberball, and during the second manipulation, allowing us to observe whether both situations—inclusion and exclusion—produced an increase in the arousal level with this measurement, as Iffland et al. (2014) identified in their study.

#### Positive Affect and Anger

For positive affect, participants recorded their answers to three items (e.g., “happy”) extracted from the short version of the Pleasantness subscale of the positive affect factor of the Positive and Negative Affect Schedule (Watson et al., 1988), presented before (α = 0.78) and after (α = 0.81) manipulation. The scale was calculated by the mean of the three items. For anger, participants also recorded their answers to three items (e.g., “angry”) extracted from the short version of the Anger subscale (Spanish version; Fernández et al., 2002) of the Profile of Mood States questionnaire (McNair et al., 1971), presented before (α = 0.79) and after (α = 0.91) manipulation. The scale was calculated by the mean of the three items. For both the positive affect and the anger scales, participants had to respond to what extent (from 1 = “not at all” to 7 “totally”) they felt “happy,” “angry,” etc., in that moment. To evaluate the change experienced between positive affect and anger, evaluated before and after manipulation, two measures were created from the differential between both moments: delta positive affect and delta anger.

#### Social Affiliation Motivation

In order to assess participants’ level of desire to be included in their task group in the future, or motivation to be affiliated with their group (α = 0.89), a three-item 7-point Likert scale ranging from 1 to 7 was created (e.g., “I would like to be fully accepted by the members of this group in the future”; see Table 1). The scale was calculated by the mean of the three items.

**TABLE 1:** Items in the social affiliation motivation scale.

1. “I would like to be fully accepted by the members of this group in the future”
2. “I would like to be fully integrated into this group in the future”
3. “I would like the members of this group to accept me in the future”

#### Skin Conductance

Galvanic skin conductance was continuously recorded throughout the experiment using a second computer connected to the biofeedback package (PHYSIOLAB Technologies, J&J Engineering I-330-C2, Milton Keynes, United Kingdom) with a constant voltage of 0.5 V (as in Cuadrado, 2015). Skin conductance levels were obtained using a skin-resistance sensor cable connected to electrodes placed on participants’ non-dominant hand and attached to the medial phalangeal surfaces of the middle and index fingers. An electrodermal gel was used as an electrolyte for conductance, and responses were displayed in microsiemens (ηS) per second. All data were recorded during the experimental session. The mean of the scores obtained before playing Cyberball (block 1 – baseline − 7.22 min: *M* = 5.26; *SD* = 1.78) and during Cyberball (block 2 – Cyberball manipulation time − 2.17 min: *M* = 6.81; *SD* = 2.44) were used to create the measure of arousal produced by the inclusion or exclusion condition of the first manipulation. Similarly, the mean of the scores obtained during the time in which participants were informed via their screen why the fictional participants had rejected or included them was used to create the measure of arousal produced by the inclusion or exclusion condition of the second manipulation (block 3 – information manipulation time − 0.33 min: *M* = 6.81; *SD* = 2.42). To evaluate the change experienced between the three measures of skin conductance over time, a first measure of skin conductance evolution (SC1) was calculate between block 2 – Cyberball and block 1-Baseline (SC1 = (SC_block2_Cyberball – SC block1_Baseline) / SC block1_Baseline); a second measure of skin conductance evolution (SC2) was calculated between block 3-Manipulation and block 2-Cyberball (SC2 = (SC_block3_Manipulation – SC block2_ Cyberball) / SC block2_ Cyberball); and lastly a third measure of skin conductance evolution (SC3) was calculated between block 3-Manipulation and block 1-Baseline (SC3 = (SC_block3_Manipulation – SC block1_Baseline) / SC block1_Baseline).

#### Prosocial Behavior

In order to measure prosocial behavior (α = 0.77), we used the Public Good Dilemma game, as in Cuadrado (2015). Some points were given to the participants (all participants had 3 points in the first round, 4 in the second round, and 6 in the third round) and every player decided how many points to donate or keep. Points donated to their group (the simulated group with which they thought they were playing in the Cyberball game) were doubled and distributed among all group members. The more they gave to the group, the less they won individually. Inversely, more selfish behavior, such as keeping all the points, was the more advantageous way to gain more money individually. They were informed that the money they would gain at the finalization of the study would depend directly on the number of points accumulated. The mean number of points donated in three rounds of the game was used as a prosocial behavior measure; thus, the more points participants gave to the group, the more prosocial they were. They were informed that the money they finally won would depend on the points accumulated in each task.

### Treatment of the Data

#### Manipulation Check

To confirm that our manipulation of inclusion and exclusion had the expected effect on participants, a t-test for independent samples was performed on our Perceptions of Exclusion Scale with SPSS 25.

#### Effect of Inclusion and Exclusion on the Study Variables

To test the impact of exclusion on skin conductance, positive affect, and anger, several *t*-tests for independent samples, one-sample-*t*-tests, and paired sample *t*-test were performed with SPSS 25.

#### Relationships Between Variables

Relationships between the variables were observed by performing a Pearson correlation analysis for each one of the two experimental groups. Moreover, linear regression analyses were performed with the global sample to explore which variables remained direct predictors for social affiliation motivation and for prosocial behavior. For social affiliation motivation, the predictive variables introduced were the perception of exclusion, the change in anger and positive affect between, after, and before the manipulation, and the evolutions of skin conductance. For prosocial behavior, social affiliation was added as a supplemental predictive variable.

Finally, a path analysis was performed with AMOS.23. The goodness-of-the-fit indices was explored by assessing the chi-squared (χ^2^), the root mean square error approximation (RMSEA), CFI (Comparative Fit Index), and the GFI (Goodness of Fit), and by applying the Schermelleh-Engel et al. (2003) rules of thumb.

## Results

### Manipulation Check

The *t*-test performed on the Perception of Exclusion Scale confirmed that our manipulation had the expected effect on participants: excluded participants (*M* = 5.75, *SD* = 1.15) perceived higher levels of exclusion [*t*(1,43) = −14.45; *p* < 0.001; Cohen’s *d* = 4.18; effect-size *r* = 0.91] than included participants (*M* = 1.76, *SD* = 0.71).

### Effect of Inclusion and Exclusion on Positive Affect and Anger

**FIGURE 1:**
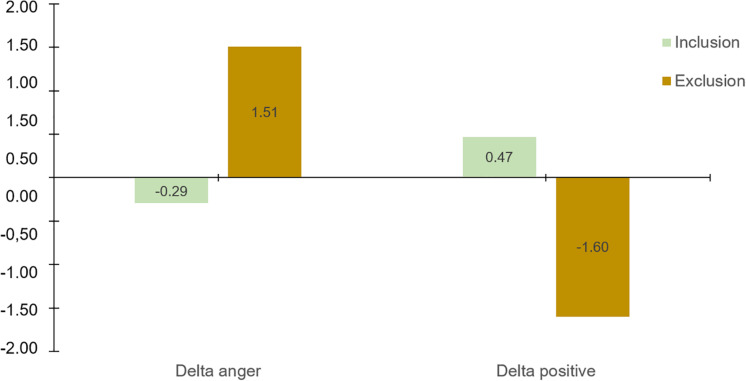
Variation of anger and positive affect in the included and excluded groups.

The *t*-tests for independent sample performed showed a significant difference [*t*(1,46) = -4.40; *p* < 0.001; Cohen’s *d* = 1.27; effect-size *r* = 0.54] in the variation of anger (Delta anger) between inclusion and exclusion. As expected, and as can be observed in Figure 1, the one-sample *t*-tests showed that there was a significant increment in anger for the excluded group [*t*(1,23) = 4.11; *p* < 0.001; Cohen’s *d* = 1.19; effect-size *r* = 0.51], but no significant variation of anger for the included one [*t*(1,23) = −1.60; *p* = 0.12; Cohen’s *d* = 0.46; effect-size *r* = 0.23].

In addition, the *t*-tests performed showed a significant difference [*t*(1,46) = 6.45; *p* < 0.001; Cohen’s *d* = 1.86; effect-size *r* = 0.68] in the variation of positive affect (Delta positive) between inclusion and exclusion. And as expected, the one-sample *t*-tests showed a significant variation of positive affect for both the included and the excluded samples: for the excluded group, a significant decrement of positive affect was found [*t*(1,23) = −6.75; *p* < 0.001; Cohen’s *d* = 1.95; effect-size *r* = 0.70]; meanwhile for the included group, a significant increase in positive affect was found [*t*(1,23) = 2.18; *p* < 0.05; Cohen’s *d* = 0.63; effect-size *r* = 0.30], as can be observed in Figure 1. Thus, Hypothesis 1a was confirmed.

Moreover, the paired sample *t*-test performed on the whole sample showed that the mean of the difference between the variation in anger (*M* = 0.61, *sd* = 1.68) and the variation in positive affect (*M* = −0.56, *sd* = 1.52) was significant [*t*(1,47) = 2.77; *p* < 0.01; Cohen’s *d* = 0.73; effect-size *r* = 0.34]. As expected in hypothesis 1b, the variation in anger was higher than the variations in positive affect.

### Effect of Inclusion and Exclusion on Skin Conductance

The paired *t*-tests performed showed (a) a significant difference for the variation in skin conductance between the baseline and the first manipulation time [*t*(1,44) = 6.90; *p* < 0.001]; (b) no difference for the variation in skin conductance between the first manipulation time and the second manipulation time[*t*(1,44) = 0.35; *p* = 0.73]; and (c) a significant difference for the variation in skin conductance between the baseline and the second manipulation time [*t*(1,44) = 6.80; *p* < 0.001]. Thus, there was a significant evolution of skin conductance between the baseline and the manipulations, but not between both manipulations.

The *t*-tests for independent sample performed did not show significant differences (a) between inclusion and exclusion in the variation of skin conductance between the baseline and the first manipulation time [*t*(1,43) = 0.60; *p* = 0.553]; (b) nor in the variation of skin conductance between the baseline and the second manipulation time [*t*(1,43) = 0.33; *p* = 0.746]; (c) nor in the variation of skin conductance between the first manipulation time and the second manipulation time [*t*(1,43) = −1.016; *p* = 0.315].

Thus, Hypothesis 2 was not confirmed.

### Relationships of Skin Conductance Evolution With Emotions and Social Affiliation Motivation, and Prosocial Behavior

The results of the correlational analyses performed by comparing the included and the excluded groups revealed the results showed in Table 2, where support was found for Hypothesis 3a and 3b (skin conductance change was related with lower levels of social affiliation motivation and prosocial behavior for excluded individuals, but not for included individuals).

**TABLE 2:**
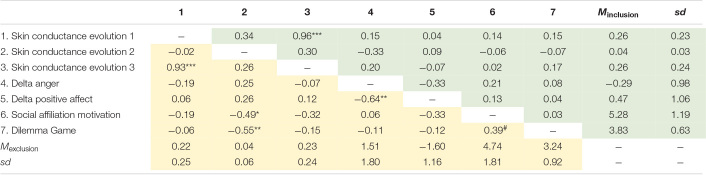
Correlation and means of the study variables.

***p < 0.001; **p < 0.01; *p < 0.05; ^#^*p* < 0.09; Skin conductance evolution 1 = Evolution of skin conductance between the baseline and the first manipulation; Skin conductance evolution 2 = Evolution of skin conductance between the first and the second manipulations; Skin conductance evolution 3 = Evolution of skin conductance between the baseline and the second manipulation; Values for the included group are shown above the diagonal and highlighted in green, and values for the excluded group are shown below the diagonal and highlighted in yellow.

Moreover, the results of the linear regression analysis with social affiliation motivation as dependent variable revealed that the model explained 15% of the variance (*R*^2^ Adj = 0.15; *F* (6,41) = 2.43, *p* < 0.05). Only the evolution of skin conductance 2, i.e., between both manipulations (ß = −0.36, *p* < 0.02) and the perception of exclusion (ß = −0.53, *p* < 0.05), acted as direct predictors of social affiliation motivation, giving additional support for Hypothesis 3a.

### Social Affiliation Motivation as a Predictor of Prosocial Behavior

The results of the hierarchical linear regression analysis revealed that the model explained 16% of the variance (*R*^2^Adj. = 0.16; *F* (6,41) = 2.32, *p* < 0.05). Only social affiliation motivation acted as a direct predictor of prosocial behavior (ß = 0.37, *p* < 0.03). The results supported the predictive role of social affiliation motivation in prosocial behavior (Hypothesis 4).

### Predictive Model of Prosocial Behavior

**FIGURE 2:**
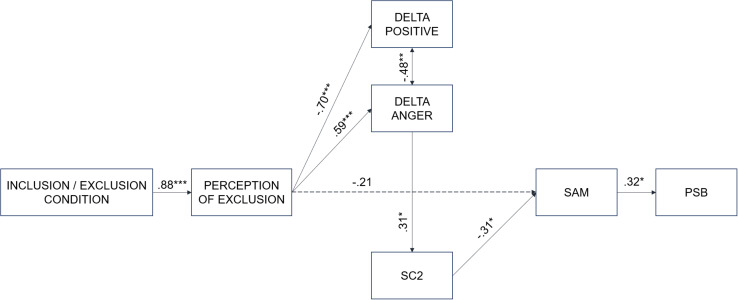
Predictive model of prosocial behavior. SC2 = variation of skin conductance; SAM = social affiliation motivation; PSB = prosocial behavior. *p < 0.05; **p < 0.01; ***p < 0.001.

Once the analyses were performed, a path analysis with AMOS 0.23 was performed to explore all the relationships of the variables in a unique model predictor of prosocial behavior. The results supported the main study hypotheses, with the model showing good fit indices (χ^2^ (13, N = 48) = 14.64, *p* = 0.330; RMSEA = 0.05 (95% confidence interval = [0.001, 158]; CFI = 0.99; GFI = 92) (Figure 2).

## Discussion

The aim of this experiment was to observe the consequences of social inclusion and exclusion experiences on moods, social affiliation motivation, and skin conductance, and to explore the possible effect of interaction between skin conductance and anger due to the inclusion/exclusion experience in the prediction on social affiliation motivation and prosocial behavior. We argued that anger produced by exclusion will enhance social affiliation motivation when individuals feel especially aroused by their exclusion (and then show high skin conductance levels), but not when the evolution of emotional arousal after exclusion is lower. Moreover, the effect of social affiliation motivation on prosocial behavior was assessed. Young people are especially affected by an inherent need to belong, and it has been argued that prosocial behavior may be a way to gain social acceptance. In this sense, young people with higher social affiliation motivation would be more predisposed to act prosocially.

### Consequences of Inclusion and Exclusion

Regarding the consequences of inclusion and exclusion on moods, the results of this study have shown that while the experience of exclusion enhances the anger levels of individuals, the experience of inclusion promotes positive moods related to pleasantness, such as happiness, in agreement with previous studies (Chow et al., 2008; Blackhart et al., 2009; Romero-Canyas et al., 2010; Cuadrado et al., 2015). Therefore, the exclusion experience seems to be painful, as other researchers have found (Eisenberger and Lieberman, 2004; Eisenberger et al., 2007, 2011; Kawamoto et al., 2012). On the contrary, the inclusion experience seems to be pleasant for included individuals (Blackhart et al., 2009). Moreover, the results have shown that the variation in anger suffered by the excluded group was higher than the variation in positive affect suffered by the included group, in accordance with previous studies (Seidel et al., 2013). Rejected individuals felt angrier than included individuals who felt positive. Therefore, the subjective emotional impact of exclusion situations appears to be stronger than the impact of inclusion situations. In agreement with previous literature, once more those results provide support for the psychological negative impact on subjective well-being and mental health of exclusion for individuals. In this regard, it has been proven that social exclusion is a very threatening situation that impairs fear acquisition and generalization, and is related to anxiety (Heeren et al., 2017; Dou et al., 2020). This fact might explain why, as a highly threatening situation, its impact on subjective negative emotion is higher than the impact that inclusion has on positive emotions.

Both inclusion and exclusion arouse individuals. But exclusion is related with fear, pain, anxiety, and stress (Eisenberger and Lieberman, 2004; Heeren et al., 2017; Dou et al., 2020); in the same way that we found a heightened impact of social exclusion on anger, in comparison with the impact of inclusion on positive affect, we expected to find a heightened impact of social exclusion on the emotional arousal of individuals (measured with skin conductance variation), in comparison with inclusion. Nevertheless, the results showed that skin conductance increased significantly after the manipulation for both the included and the excluded sample, but no differences were found between the two groups. Therefore, our results were not able to demonstrate that an exclusion situation produces higher arousal than an inclusion one, but supported the idea that both exclusion and inclusion situations produce arousal in individuals, arousal that in the situation of inclusion may be due to an activation of heightened positive affect, and in the situation of exclusion to an activation of heightened negative affect. It should be interesting for future studies with larger samples and with a control and experimental group to explore the possible relation of social exclusion and inclusion and an increase in skin conductance.

Our results supported the expected relationship between skin conductance and social affiliation motivation. Excluded individuals with higher skin conductance variation felt less motivated to affiliate with their peers. Nevertheless, this pattern was not observed in included individuals. These results are congruent with the findings of Maner et al. (2007) who show that excluded individuals are not motivated to reconnect with individuals who have rejected them. In addition they are in agreement with the results found by Serafini et al. (2017), who showed that individuals highly sensitive to rejection tend to produce exaggerated negative emotions and depression. Moreover, these results are also congruent with previous research works stating that the desire to reconnect does not arise in included individuals, as their inclusion is already secure (Cuadrado, 2015).

Furthermore, as expected, our results have shown that, for excluded individuals, high skin conductance variations are linked to lower levels of prosocial behavior. Nevertheless, no relation was observed between prosocial behavior and skin conductance in included individuals. These results are congruent with the results found by Hein et al. (2011), in which they argued that skin conductance levels are linked to empathy and correlates with the tendency to prevent other to feel pain. With excluded individuals, an inverse pattern is observed; a high arousal after exclusion would be related with lowered intention to connect with those who have rejected them, and consequently a lower desire to help them. In this sense, other studies have suggested that the increase on physiological arousal correlates with reactive aggressive behaviors that are characterized as “hot blooded”. Further studies on interactions between inclusion/exclusion, physiological arousal and prosocial/antisocial behavior (Raine et al., 2006; Armstrong et al., 2019) with heterogeneous and bigger samples would be desirable.

### Social Affiliation Motivation as a Predictor of Prosocial Behavior

Nonetheless, skin conductance change was found to be related with lower levels of social affiliation motivation and prosocial behavior. The more intensively individuals react to exclusion, the less they want to affiliate with other individuals who have rejected them previously, and the less they tend to behave in a prosocial way. Those results are particularly relevant because, to date, as far as we know, few studies have explored the relation between physiological responses and social affiliation motivation or prosocial behavior (see one example for preschool children population in Zahn-Waxler et al. (1995). The fact that the intensity of the affective reaction, in terms of physiological arousal, has a significant impact on affiliation is relevant and has practical implications. With the emerging portable and noninvasive technologies able to monitor the emotional state of users in different contexts, it would be easy to record the arousal level of the individuals. The implementation of these techniques in applications where social behavior is essential can take these results into consideration. Thus, the system could detect affective states and estimate the subsequent effects on social behavior and regulate them properly under specific application. For instance, while driving, where a feeling of anger is often elicited and a prosocial behavior can be desirable, the integration of arousal may be suitable to determine when emotion regulation is needed (Béquet et al., 2020).

Belonging and the maintenance of social bonds are fundamental needs for humans (Baumeister and Leary, 1995) to such an extent that, when missing, individuals attempt to achieve this inclusion by behaving prosocially (Baumeister and Leary, 1995; Mlcak and Zaskodna, 2008). Regarding individuals who are highly motivated to become affiliated with the group, behaving prosocially may be a way to achieve inclusion (Baumeister and Leary, 1995; Maner et al., 2007; Mlcak and Zaskodna, 2008) by showing that they are friendly and valuable to the group. Our results confirm this affirmation by showing that social affiliation motivation is a direct predictor of prosocial behavior. The more young people are motivated to become affiliated with the group, the more they behave prosocially.

### Limitations

Although this study has implications regarding the different impact of exclusion/inclusion experiences, it is necessary to highlight its limitations. A larger and more heterogeneous sample—particularly with respect to gender—would have been advantageous. Data were obtained from a sample of students composed exclusively of women. Although, according to the results of Seidel et al. (2013), no significant differences were found in the cortisol values recorded in men or women after experiencing a situation of social exclusion. Therefore, there is no reason to believe that the findings of this study differ by gender, although the results should be interpreted with caution and may not be generalizable to the general population. Closely related to this is the small sample size, which implies that caution must be taken when generalizing the results. Regardless, it would be interesting to replicate this study with a larger and more heterogeneous sample.

Another limitation refers to the ad hoc construction of the social affiliation motivation scale. Meanwhile, Cronbach’s alpha for the scale is encouraging and further studies should provide more evidence for its validity.

On the other hand, with regard to the effect of inclusion and exclusion on skin conductance, this remains unsecured; as we do not possess any control condition, there is no way to ascertain that either exclusion or inclusion increased skin conductance. However, future research should be replicated with a control condition to ensure that the increase is due to[ the manipulation.

In future investigations, it would be interesting to explore other variables that have been shown to influence social affiliation motivation and may differ in excluded compared to included individuals. Investigating other physiological parameters as predictors of social affiliation motivation and prosocial behavior would also be useful.

## Conclusion

In brief, the main findings of this study arise from the fact that individuals who were over-aroused by exclusion demonstrated higher social affiliation motivation, a variable that directly predicts prosocial behavior: Skin conductance reactivity—an adequate measure of the emotional activation of individuals (Khalfa et al., 2002)—directly influences social affiliation motivation. Young people who felt angry after their exclusion experience and who were intensively over-aroused by exclusion showed a decrease in their desire to be included by individuals who had rejected them. Furthermore, the results have confirmed that, in turn, young people who are highly motivated to become affiliated with a group tend to be more prosocial with the group. These results seem to support the hypothesis that prosocial behavior may be seen by individuals as a way to gain social acceptance.

## Data Availability Statement

The raw data supporting the conclusions of this article will be made available by the authors, without undue reservation.

## Ethics Statement

The studies involving human participants were reviewed and approved by Andalusian Health Service’s Research Ethics Committee and the Reina Sofía Hospital in June 2015 (Acta 242, ref 2886, 29/06/2015). The patients/participants provided their written informed consent to participate in this study.

## Author Contributions

CT and EC performed conceptualization and visualization. EC, CT, and AH-M performed methodology and supervision. CT, EC, AH-M, RC-M, and BL performed validation and writing review and editing. EC and AH-M performed formal analysis. RC-M, EC, CT, and BL performed investigation. CT performed resources, project administration, and funding acquisition. EC and RC-M performed data curation. EC performed writing original draft preparation. All authors have read and agreed to the published version of the manuscript.

## Conflict of Interest

The authors declare that the research was conducted in the absence of any commercial or financial relationships that could be construed as a potential conflict of interest.
